# A feasible strategy to balance the crystallinity and specific surface area of metal oxide nanocrystals

**DOI:** 10.1038/srep46424

**Published:** 2017-04-24

**Authors:** Q. P. Zhang, X. N. Xu, Y. T. Liu, M. Xu, S. H. Deng, Y. Chen, H. Yuan, F. Yu, Y. Huang, K. Zhao, S. Xu, G. Xiong

**Affiliations:** 1 Key Laboratory of Information Materials of Sichuan Province & School of Electrical and Information Engineering, Southwest University for Nationalities, Chengdu, 610041, China; 2 Plasma Sources and Application Center, NIE, and Institute of Advanced Studies, Nanyang Technological University, 637616, Singapore; 3 Department of Chemistry, University of Cambridge, CB2 1EW, Cambridge, UK

## Abstract

Practical, efficient synthesis of metal oxide nanocrystals with good crystallinity and high specific surface area by a modified polymer-network gel method is demonstrated, taking ZnO nanocrystals as an example. A novel stepwise heat treatment yields significant improvement in crystal quality. Such nanophase materials can effectively degrade common organic dyes under solar radiation and can perform very well in photo-assisted detection of NO_2_ gas. Other typical metal oxide nanocrystals with good crystallinity and high specific surface area were also synthesized successfully under similar conditions. This work provides a general strategy for the synthesis of metal oxide nanocrystals, balancing the crystallinity and specific surface area.

Environmental pollution and destruction has gained the attention of the whole society and government because of its harmful influences on human health and sustainable development of society. It makes waste-water treatment^1^,^2^ and detection of toxic and hazardous gases^3^,^4^ more and more important to humans. The “advanced oxidation process” (AOP), based on heterogeneous photocatalysis, shows great potential in waste-water treatment compared with traditional techniques, since it is highly efficient, inexpensive and environment-friendly, and produces no secondary pollution^5^. A typical photocatalytic system requires materials that have an ideal bandgap to effectively harvest a large portion of the solar spectrum, and they must have suitable conduction and valence band edges for targeted reactions. At the same time, these materials should be abundant, easily accessible and stable in the long term. Semiconductor materials, especially metal oxides, meet these requirements well.

On the other hand, the photocatalytic activities and gas sensitivities are enhanced by using materials with high specific surface area, which provides abundant reactive sites for increasing the adsorption of target species^6^,^7^. Some general strategies, such as decreasing particle sizes to nanoscale^8^ or designing corresponding two-dimensional nanosheet forms^9^,^10^, are employed to increase the surface area. Many nano-metal oxides have been reported as good photocatalysts – e.g. titanium dioxide (TiO_2_)^11^, zinc oxide (ZnO)^12^, hematite (α-Fe_2_O_3_)^13^, zirconium dioxide (ZrO_2_)^14^, tin dioxide (SnO_2_)^15^, cerium dioxide (CeO_2_)^16^. In recent years, inspired by research in the photocatalysis and photodetection field, researchers found that nanoscale ZnO, SnO_2_ and TiO_2_ exhibit good gas sensing property at room temperature with UV illumination^17^,^18^,^19^. Nanoscale metal oxide semiconductors (MOSs) have been widely identified as promising materials for use in environmental remediation and monitoring. However, bulk defects can be introduced during nanostructure formation, which usually act as recombination centers for photogenerated electron-hole pairs, encumbering the otherwise superior photoactivity of nanoscale MOSs^20^. To overcome this impediment, improving the crystal quality of nanoscale MOSs is critically important. Optimization of crystallization temperature and time is a common method that can significantly reduce the number of defects, but as crystallization continues, increasing particle size decreases the specific surface area^21^. Therefore, optimizing synthesis and crystallization processes is essential to balancing good crystallinity and high specific surface area^22^, which results in high photocatalytic and gas-sensing performance of nanoscale MOSs. To date, developing such balancing techniques remains a tremendous challenge.

In the present work, we have developed a simple and effective strategy (Fig. 1) to synthesize MOS nanocrystals with both good crystallinity and high specific surface area. It combines a modified polymer-network gel process and a novel stepwise heat treatment process. The former is a multistep reaction process, involving metal ion chelation and acrylamide polymerization, promoting homogeneous distribution of metal precursors in aqueous solution at a molecular level and preventing aggregation of gel particles. The latter guarantees a thorough release of thermal stress during the annealing of xerogel, consequently optimizing crystallization. As an example, the morphological evolution of ZnO nanocrystals obtained using different heat treatments demonstrates the basis of high-quality nanocrystals formation. We found that the most efficient photocatalytic decomposition of organic pollutants was obtained with ZnO nanocrystals prepared by using this novel strategy, due to their good crystallinity and high specific surface area. These nanocrystals also show excellent performance in photo-assisted detection of NO_2_ gas.

## Results

### Well crystallized ZnO nanocrystals with high surface areas

**Figure 1:**
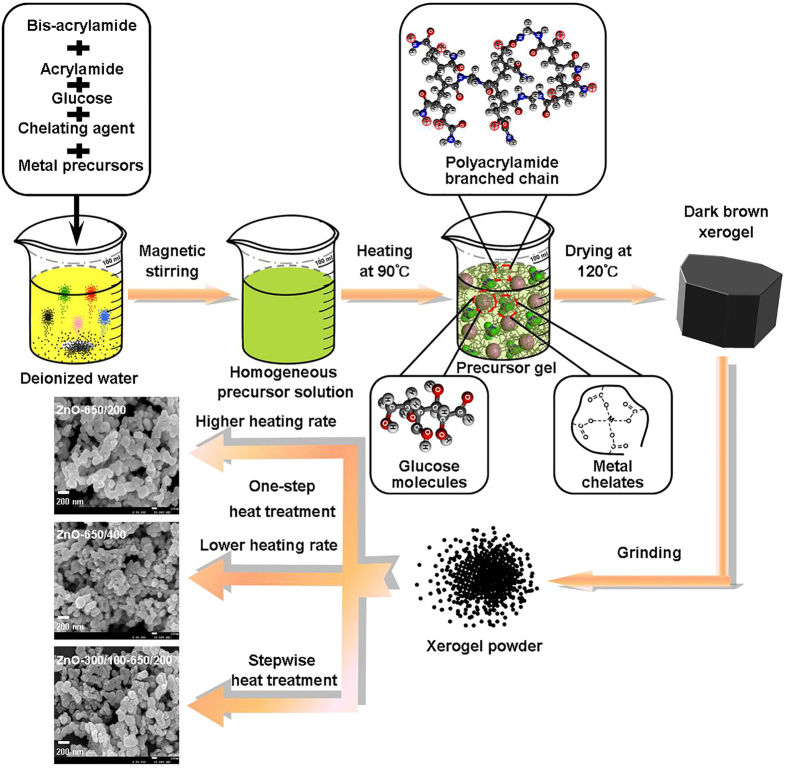
Schematic of fabrication of well crystallized metal oxide nanocrystals with high specific surface area. FE-SEM images of ZnO nanocrystals obtained using different heat treatments appear in the lower left corner.

Our strategy, combining a modified polymer-network gel process and a novel stepwise heat treatment process, to fabricate MOS nanocrystals is illustrated in Fig. 1. The metal precursors, chelating agent, glucose, acrylamide (AM) and bis-acrylamide (MABM) are added to deionized water one by one to form a transparent solution at room temperature. Then the whole solution is heated to 90 °C and the temperature is kept for a few minutes under magnetic stirring. During this process, the chelation of metal ions and polymerization of acrylamide are essential for formation of the nanocrystals. The precursor gel shown in Fig. 1 is formed by mutual nesting between chelated metal, branched polyacrylamide chain and glucose molecules. These three compounds are bound together through weak hydrogen bonds/electrostatic interactions. As the chelating agent prevents the uncontrolled hydrolysis reaction, this gel process allows metal ions to coordinate with polymers, resulting in a homogeneous distribution of metal precursors in aqueous solution at a molecular level^23^,^24^. A tangled polyacrylamide network then forms as the polymerization reaction of AM and MABM monomers is initiated by high temperature (avoiding using a trigger agent such as ammonium persulfate), which reduces aggregation of the chelate for better homogeneity. A large number of glucose molecules fill in the spaces of the polymer network and prevent the network from collapsing rapidly during the drying process. After that, a dark brown xerogel, obtained by drying the precursor gel at 120 °C for 24 h in a thermostat dryer, is ground into a fine powder in an agate mortar. Finally, the evenly distributed MOS nanocrystals form as the polymers decompose, when treated at a desired temperature. During this process, heat treatment can influence the crystallization process of nanocrystals, and optimizing the heat treatment process is a feasible way to further improve their crystal quality. Based on thermoanalysis, the stepwise heat treatment route is employed in this study, aiming to obtain better quality MOS nanocrystals and enhanced photocatalytic and gas-sensing performance.

**Figure 2:**
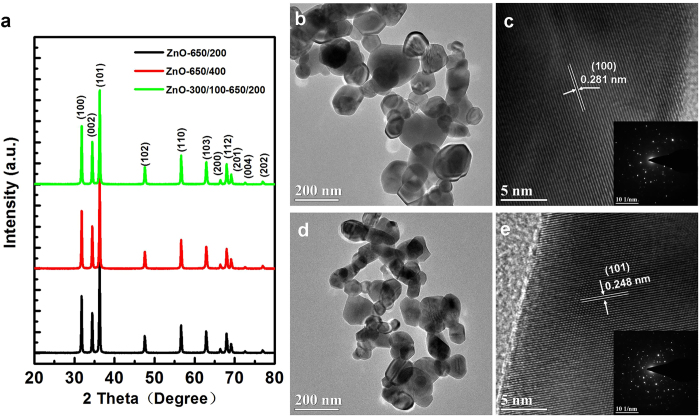
Crystal properties and morphology of different ZnO nanocrystals. (**a**) XRD patterns of ZnO nanocrystals obtained using different heat treatments. (**b**,**d**) TEM images and (**c**,**e**) high-resolution TEM images of ZnO-650/400 samples and ZnO-300/100–650/200 samples; insets are the corresponding SAED patterns. Polycrystalline characteristic of wurtzite ZnO phase can be confirmed by the SAED patterns.

To demonstrate the effectiveness of this strategy, three samples of ZnO nanocrystals were synthesized by a modified polymer-network gel process followed by different heat treatment processes (see Fig. 1 and Methods), denoted as ZnO-650/200, ZnO-650/400 and ZnO-300/100–650/200, respectively, where ZnO-650/400 is a reference for comparison. In synthesis of ZnO nanocrystals, tartaric acid (TA) is chosen as chelating agent, which serves as a hydroxy carboxylic acid for chelation of metal ions^25^. The heat treatment strategy for synthesis of ZnO-300/100–650/200 was devised based on thermogravimetric (TG) and derivative thermogravimetric (DTG) analyses of ZnO xerogel powder (Supplementary Fig. 1). The powder X-ray diffraction (XRD) patterns of different ZnO nanocrystals are shown in Fig. 2a. All the diffraction peaks can be indexed to the wurtzite phase of ZnO (JCPDS file No. 36-1415). No obvious impurity peaks were found. The strong and sharp XRD diffraction peaks mean that the ZnO nanocrystals were well crystallized. The average crystal sizes of ZnO-650/200, ZnO-650/400 and ZnO-300/100–650/200 are determined to be 42.13, 42.40 and 41.40 nm, respectively, according to the Scherrer formula (D = 0.89*λ*/*β*cos*θ*) (Table 1). The field emission scanning electron microscopy (FE-SEM) images in Fig. 1 reveal that all the ZnO nanocrystals are irregular and multilateral in shape and have a wide range of particle sizes (50 nm <diameter <200 nm). ZnO-300/100–650/200 and ZnO-650/400 have marginally smaller average particle sizes than ZnO-650/200, while there is a certain degree of particle aggregation in ZnO-650/400, and the surface profile is more distinct for ZnO-300/100–650/200 than for the other ZnO nanocrystals. Figure 2b–e show transmission electron microscopy (TEM) images, high-resolution TEM (HRTEM) images and corresponding selected area electron diffraction (SAED) patterns of ZnO-650/200 and ZnO-300/100–650/200, respectively. As illustrated in the insets of Fig. 2c and e, both ZnO samples show polymorphic behavior. The clearer lattice fringes in the HRTEM image (Fig. 2e) show that the crystallinity of ZnO-300/100–650/200 is better than that of ZnO-650/200. The lattice fringe spacings of 0.281 nm for ZnO-650/200 and 0.248 nm for ZnO-300/100–650/200 are in line with those of the (100) and (101) planes of hexagonal ZnO (Fig. 2c and e), respectively.

The above observations indicate that the as-prepared ZnO nanocrystals are composed of polycrystalline particles, aggregates of several single crystals, and ZnO-300/100–650/200 exhibits both the smallest average particle size and the best crystal quality. These superior qualities are due to the use of the novel stepwise heat treatment process in which ZnO xerogel powder was pre-calcinated at 300 °C for 100 min before being recalcinated at 650 °C for 200 min (see Methods). In comparison with traditional annealing processes (one-step heat treatment process), such special heat treatment is a suitable strategy to guarantee a thorough release of thermal stress, which effectively reduces the aggregation of particles so that more single-crystal ZnO nanoparticles form with continuous and ordered interior crystal structure. The Brunauer-Emmett-Teller (BET) measurement shows the specific surface areas of ZnO-650/200, ZnO-650/400 and ZnO-300/100–650/200 are 29.97, 17.61 and 29.35 m^2^/g, respectively (Table 1). It is quite clear that stepwise heat treatment does not reduce the specific surface area of ZnO nanocrystals, effectively tuning the balance between crystallization and specific surface area. Longer calcination time can improve the crystallinity of the nanocrystals^20^ but simultaneously increases aggregation, which means simply reducing the heating rate does not prevent the specific surface area from decreasing.

### Synthesis and characterization of other metal oxide nanocrystals

**Figure 3:**
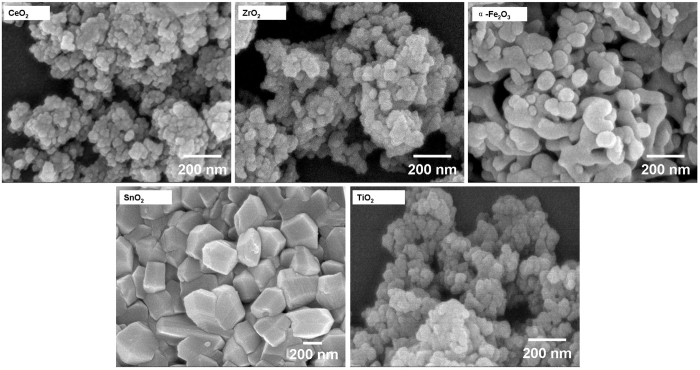
FE-SEM images of other metal oxide nanocrystals. All nanocrystals were prepared in ways similar to the synthesis of ZnO-300/100–650/200.

Other high quality metal oxide nanocrystals were prepared under conditions similar to the synthesis of ZnO-300/100-650/200 described above – CeO_2_, ZrO_2_, α-Fe_2_O_3_, SnO_2_ and TiO_2_. Figure 3 are the FE-SEM images of these nanocrystals and indicates their good uniformity. The α-Fe_2_O_3_ sample has smooth spherical particles containing some nanorods and is about 100 nm diameter; the SnO_2_ has irregular blocky-shaped particles with smooth surfaces, about 200 nm diameter; while the CeO_2,_ ZrO_2_ and TiO_2_ nanocrystals all have a round shape with almost consistent particle size around 40 nm. In CeO_2_ nanocrystals, every particle is found to be an aggregate of small crystallites. The corresponding power XRD patterns are presented in Supplementary Fig. 3, identifying these nanocrystals as the fluorite cubic phase of CeO_2_ (JCPDS No. 65-5923), monoclinic/tetragonal mixed phase ZrO_2_ (JCPDS No. 65-1022 and 50-1089), rhombohedral phase of Fe_2_O_3_ (JCPDS No. 33-0664), tetragonal rutile phase of SnO_2_ (JCPDS No. 41-1445), and anatase/rutile mixed phase TiO_2_ (JCPDS No. 65-5714 and 65-1119), respectively. It can be seen that the products are of pure crystal phase with no unidentified peaks, and the strong XRD diffraction peaks indicate that they have good crystallinity. In particular, high specific surface areas were obtained: 60.05 m^2^/g for CeO_2_, 39.15 m^2^/g for ZrO_2_, 47.30 m^2^/g for α-Fe_2_O_3_, 32.92 m^2^/g for SnO_2_, and 71.04 m^2^/g for TiO_2_ (Table 1). Supplementary Table 1 is a comparison between the nanocrystals prepared by this strategy and those by other reported methods, demonstrating its great advantage. This strategy provides a feasible way to prepare well crystallized metal oxide nanocrystals with high surface areas at relatively low cost and on large scale.

### Surface compositions of ZnO nanocrystals

**Figure 4:**
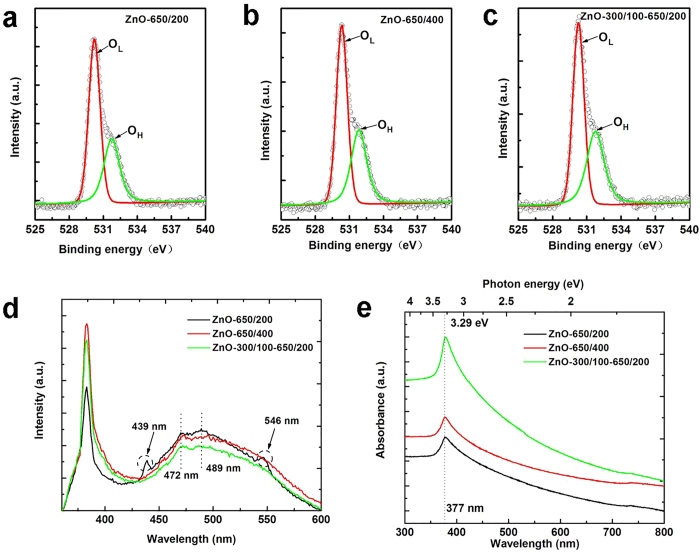
Surface composition and optical characteristics of as-prepared ZnO nanocrystals. (**a**–**c**) XPS spectra of O *1* *s* states of ZnO nanocrystals obtained using different heat treatments. (**d**,**e**) PL spectra (excited at 325 nm) and UV-Visible absorption spectra of ZnO nanocrystals obtained using different heat treatments.

There is evidence that the surface properties of nanoscale materials directly determine their catalytic activities^26^,^27^,^28^. So providing a good surface characterization for nanocrystals is very important in obtaining in-depth, comprehensive understanding of the physical and chemical processes that underlie applications in photocatalytic and gas-sensing reactions. X-ray photoelectron spectroscopy (XPS) and Fourier transform infrared spectroscopy (FTIR) analysis are frequently used to study surface structures of materials for these applications. The XPS survey spectrum (Supplementary Fig. 4a) reveals no impurity elements in any of our ZnO nanocrystal samples except Zn, O, and C (extraneous contamination), which is completely consistent with the XRD results. The Zn 2p spectra in Supplementary Fig. 4b shows the presence of Zn^2+^ lattice ions in all ZnO nanocrystals, and more O atoms were bound to Zn atoms for ZnO-650/200. The high-resolution O 1 s core-level spectra of three samples show only small differences (Fig. 4a–c). From Fig. 4a–c, it can also be seen that the O 1 s XPS spectra of all the samples are quite asymmetric, which indicates that various chemical states of oxygen exist on the surface of these ZnO nanocrystals. Based on Gauss multi-peak fitting, these broad spectra can be decomposed into two peaks located at 530.3 ± 0.1 eV and 531.8 ± 0.1 eV. The low binding energy component is typically assigned to O^2−^ ions (O_L_) in the wurtzite structure of hexagonal Zn^2+^ ions array^29^,^30^. The other, higher binding energy component is associated with loosely bound oxygen (O_H_) caused by surface hydroxyl (OH) group or O^2−^ ions in the oxygen deficient regions^31^,^32^. This is further evidenced by the presence of a broad characteristic IR adsorption peak between 3200 and 3600 cm^−1^ arising from OH stretching vibration (Supplementary Fig. 5a, highlighted by the blue-green rectangle)^33^,^34^. The as-synthesized samples exhibit very weak IR adsorption peaks centered at 2350 cm^−1^, indicating the presence of C = O residues, which are probably derived from CO_2_ in the air^35^. The strong absorption features in the range of 400–650 cm^−1^, with obvious differences, are assigned to stretching vibration of Zn-O bonds in ZnO nanocrystals^36^. A significant blue shift is observed in these Zn-O vibration absorption peaks (Supplementary Fig. 5a, ZnO-650/200 → ZnO-300/100–650/200), which we attribute to surface defects weakening the Zn-O band strength^31^.

In general, more OH groups (or H_2_O molecules) are found on the surface of non-stoichiometric ZnO_1−x_ nanocrystals, because dissociation and molecular absorption of water takes place on defect sites associated with oxygen vacancies^37^,^38^. Surface oxygen vacancies, having low formation energy and high adsorption energy, facilitate water dissociation^39^. The process occurs via proton transfer to a neighboring bridging oxygen atom, yielding two OH groups per initial vacancy^40^,^41^. In addition, oxygen vacancies in the surface of ZnO nanocrystals can accelerate the adsorption of other molecules such as O_2_, CO_2_, NO_2_ and alcohols, which considerably improves the nanocrystals’ catalytic activity and gas-sensing property^42^,^43^,^44^,^45^,^46^. Surface oxygen vacancies are therefore the main reason for adsorption of oxygen-containing species on ZnO nanocrystal surfaces, and variations in the concentration of these defects can be reflected indirectly by the intensity of the peaks related to OH and C = O.

Combining the FTIR and XPS characterizations, one can conclude that a certain amount of oxygen vacancies is successfully introduced on the surface of our ZnO nanocrystals. More details of the XPS analysis are listed in Supplementary Table 2. The calculated O_L_/O_H_ ratios are 1.54, 1.42 and 1.32 for ZnO-650/200, ZnO-650/400 and ZnO-300/100–650/200, respectively, and the Zn/O_L_ ratio is above 1 for all the catalysts, which further signifies the presence of oxygen deficiencies on these nanocrystal surfaces and shows that their oxygen vacancy concentrations rank in the order of ZnO-300/100–650/200 > ZnO-650/400 > ZnO-650/200. This shows the density of surface oxygen vacancies on ZnO nanocrystals varies with annealing route. Importantly, the stepwise heat treatment is beneficial for the formation of surface oxygen vacancy.

### Effects of defects on optical properties of ZnO nanocrystals

Photoluminescence (PL) spectroscopy analysis is a powerful characterization method for evaluating structural defects and optical quality of semiconductor materials. In all cases, the room-temperature PL spectra (Fig. 4d) of our samples consist of a strong and sharp UV emission resulting from the recombination of the free excitons of ZnO^47^ and a broad visible band due to native defects^48^. The UV emissions are stronger than the visible emissions, and the *I*_UV_/*I*_Vis_ (defined as the relative intensity of UV emission to the visible emission) ratio varies from 1.51 to 2.47 to 2.63 for ZnO-650/200, ZnO-650/400, ZnO-300/100–650/200, respectively. The higher *I*_UV_/*I*_Vis_ values for the ZnO nanocrystals obtained by reducing heating rate or using the stepwise heat treatment indicate lower concentrations of defects^49^,^50^, which is an aspect of good crystalline quality. This is consistent with the SEM and TEM observations as discussed above.

In the broad visible band, multiple peaks are superposed at different positions, indicating the presence of different types of defects in these nanocrystals. The blue luminescence at 439 nm is caused by the transitions of excited electrons from the level of zinc interstitials (Zn_i_, a shallow donor defect, mainly distributed in the interior of the ZnO crystals) to the valence band^51^. The blue-green emissions at 472 nm and 489 nm are attributed to the singly ionized oxygen vacancies (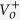) on the ZnO surface and originate from the radiative recombination of a photo-generated hole with an electron occupying the oxygen vacancy^52^,^53^,^54^. The 546 nm green emission implies that grain boundary-induced depletion regions lead to the formation of a deeply trapped doubly charged oxygen vacancy (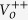) state which undergoes radiative recombination with a conduction band (CB) electron^32^. Though the origin of the visible emissions remains a matter of debate, it should be noted that all luminescence originates from the recombination of photogenerated electron-hole pairs, and providing rich information about the efficiency of charge carrier separation and trapping.

As the heat treatment process is changed, the blue (439 nm) and green (546 nm) PL peaks for ZnO-650/400 and ZnO-300/100–650/200 vanish, unlike the PL spectra for ZnO-650/200. The intensity of the blue-green (472 nm and 489 nm) PL peaks also varies with different ZnO nanocrystals, ranking as: ZnO-650/200 > ZnO-650/400 > ZnO-300/100–650/200. Interestingly, the order contrasts sharply with the ranking of surface oxygen vacancy concentration (per XPS and FTIR results), suggesting that the larger the concentration of surface oxygen vacancies, the weaker the PL peak intensity and the greater the separation of photogenerated electron-hole pairs. On the basis of the above discussion, we can be confident that defect concentration and spatial location play vital roles in the efficiency of charge carrier separation. Bulk defects, Zn_i_ and oxygen vacancies at ZnO-ZnO grain boundaries, can increase the probability of charge carrier recombination. In contrast, carrier recombination can be prevented by surface oxygen vacancies, because these defect sites easily trap electrons and energetically adsorb donor and acceptor reagents that could otherwise react with holes and electrons, giving rise to a great number of reactive oxygen species (ROS). Highly efficient charge carrier separation favors superior catalytic and gas-sensing performance under light irradiation.

From another point of view, ZnO nanocrystals with better crystallization or lower C_bulk_/C_surface_ ratio (the concentration of bulk defects relative to surface defects), exhibit more prominent UV emission in PL spectra, meaning that they are capable of greater optical absorption. To verify this, we carried out UV-Vis absorption spectroscopic analysis for all the nanocrystal samples; the results appear in Fig. 4e. As expected, the strongest absorption band at 377 nm is obtained for ZnO-300/100–650/200. However, UV absorption of ZnO-650/400 and ZnO-650/200 were very similar, which is not in compliance with the evolution of the crystallization or the ranking of C_bulk_/C_surface_. Other factors can govern optical absorption, such as specific surface area. (The specific surface area of ZnO-650/400 is smaller than that of ZnO-650/200 (Table 1).) High specific surface area provides more effective contact area for incident photons, enhancing optical absorption. The spectral response range of ZnO depends on its bandgap energy (E_g_), which can be calculated from the equation E_g_ = 1240/λ^55^, where E_g_ and λ are the bandgap energy (eV) and the wavelength (nm). No variation was found in the bandgap values among our ZnO nanocrystals – all are 3.29 eV. The consistency of these values indicates that the spectral response range of ZnO has not been extended significantly into the visible light range, because the low concentration of surface oxygen vacancies is not enough to cause disorder in the surface layers of nanophase ZnO^30^. This result is supported by the fact that all our ZnO nanocrystals are white.

### Photocatalytic performance of ZnO nanocrystals

**Figure 5:**
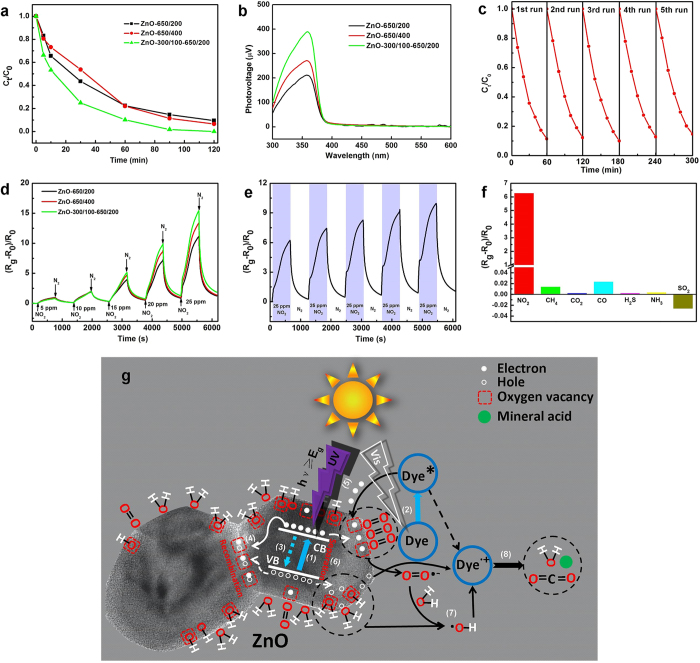
Photocatalytic & gas-sensing performance of as-prepared ZnO nanocrystals. (**a**) Photodegradation of MO over ZnO nanocrystals under simulated sunlight irradiation. (**b**) SPV spectra of ZnO nanocrystals obtained using different heat treatments. (**c**) Cycling runs for photodegradation of MO over ZnO-300/100–650/200 under simulated sunlight irradiation. The normalized concentration of MO aqueous solution is monitored by measuring the absorbance at 464 nm. (**d**) Real-time response curves of sensors based on different ZnO nanocrystals to NO_2_ with concentrations ranging from 5 to 25 ppm under 365 nm light irradiation at room temperature. (**e**) Reproducibility of sensor performance based on ZnO-300/100–650/200 response to 25 ppm NO_2_ under 365 nm light irradiation at room temperature. (**f**) Selectivity of the sensor based on ZnO-300/100–650/200 with presence of interference gases (25 ppm) under 365 nm light irradiation at room temperature. (**g**) Schematic of the catalytic reaction mechanism of ZnO-300/100–650/200 under simulated sunlight irradiation: (1) Generation of electron-hole pairs under UV irradiation, (2) dye molecules’ excitation by visible light, (3) recombination of photo-generated electrons and holes in the bulk, (4) recombination of photo-generated electrons and holes caused by grain-boundary defects, (5) surface oxygen vacancy-induced separation of electron-hole pairs, (6) electron injection from excited dye molecules into the surface and CB of ZnO, (7) formation of ROS, (8) degradation of dye molecules.

Photocatalytic reactions are determined primarily by three reaction processes: light-harvesting processes; charge generation and separation processes; and catalytic reaction processes^56^. The former two processes have a close relationship with the catalytic property itself, unlike the latter, which is affected mainly by external factors such as the reaction temperature and pH, active solution versus catalyst concentration and so on. In the present work, all such external factors were eliminated in the comparison tests for dye photodegradation, and the measured conditions were kept the same. Figure 5a shows the photocatalytic activity of different ZnO nanocrystals, as determined by monitoring the photodegradation of methyl orange (MO) under simulated sunlight irradiation. (*C*_t_/*C*_0_ is used to describe the degradation, where *C*_0_ is the initial concentration of dyes before illumination and *C*_t_ is the residual concentration of dyes at time *t*.) As depicted in Fig. 5a, ZnO-300/100–650/200 shows the highest photocatalytic activity, while those for ZnO-650/400, ZnO-650/200 differ little from each other. After 30 min, the degradation efficiencies for ZnO-300/100–650/200, ZnO-650/400 and ZnO-650/200 are 75.1%, 46.3% and 56.5%, respectively. After 120 min, MO is completely decomposed with ZnO-300/100–650/200, while ZnO-650/400 and ZnO-650/200 decompose only 93.4% and 90.5% of MO, respectively. To compare the photocatalytic performance of catalysts further, the plots of ln(*C*_0_/*C*_t_) versus time were depicted using a linear fitting method. It turned out that the degradation process fits a pseudo-first-order kinetic model. The calculated apparent rate constants (*k*) shown in Supplementary Fig. 6 are 0.04156, 0.02279 and 0.01937 min^−1^ for ZnO-300/100–650/200 and ZnO-650/400 and ZnO-650/200, respectively. Considering these results, the photocatalytic efficiency of ZnO nanocrystals is improved by 1.18 or 2.15 times by reducing heating rate or employing the stepwise heat treatment process, respectively. A similar improvement occurs in the photocatalytic decomposition of Rhodamine B (RhB) (Supplementary Fig. 8). The significant improvement of photocatalytic efficiency is attributed to a considerable enhancement in the efficiency of separating photogenerated charge carriers, arising from a reduced C_bulk_/C_surface_, as a result of the improved crystalline quality. Surface photovoltage (SPV) spectra (Fig. 5b) were obtained to further confirm the above conclusion, the corresponding surface photocurrent (SPC) spectra are shown in Supplementary Fig. 7. It can be seen that the SPV peaks of all the ZnO nanocrystal samples are located at 359 nm with a response threshold of 385 nm, which can be attributed to electron transitions from the valence band to the conduction band (O_2p_ → Zn_3d_)^57^. Their variation in intensity is consistent with their photocatalytic activity ranking: ZnO-300/100–650/200 > ZnO-650/400 > ZnO-650/200. The stronger SPV response indicates more efficient separation of photogenerated electron-hole pairs, resulting in higher photocatalytic efficiency. Apart from the response peaks, no other SPV response is observed in the visible light region, consistent with the UV-Vis absorption behavior. This demonstrates that visible light cannot induce effective separation of photogenerated electron-hole pairs in these materials; that is, the catalysts themselves might not possess photocatalytic ability in the visible spectrum.

To understand the photocatalytic mechanism better, photodegradation of MO, RhB and methylene blue (MB) were also undertaken with ZnO-300/100–650/200 under visible light irradiation (λ > 400 nm). The photodegradation rate constants of these dyes, for comparison, are shown in Supplementary Fig. 9. Their photodegradation includes two simultaneous processes: photocatalytic decomposition by ZnO nanocrystals and photosensitization related to the surface-adsorbed dye molecules. RhB and MB are dye-photosensitizers that strongly absorb visible light. When they are excited by visible light, the excited electrons immediately inject into the surface and CB of ZnO (paths 2 and 5 in Fig. 5g) and take part in the catalytic reaction processes. MO is known to be a difficult-to-degrade azo dye, wherein negligible degradation is observed, most probably because its absorption ability is weaker in the visible light range, leading to rapid recombination of excited electrons with holes. In the case of simulated-sunlight induced photodegradation, ZnO-300/100–650/200 exhibits very high degradation efficiency. Its degradation rates for MB and RhB are about 4 and 13 times higher than under visible light irradiation, respectively, indicating that UV irradiation is indispensable for a photodegradation system with the help of photosensitization of dyes.

### Gas-sensing performance of ZnO nanocrystals under UV irradiation

Light-activated metal oxide-based gas sensors have attracted considerable interest due to their potential for high sensitivity at room temperature^18^,^19^. When ZnO is exposed to UV energy near its bandgap, the strong photoconduction response makes equally favorable adsorption-reaction-desorption behavior possible at room temperature. Figure 5d shows the responses of sensors based on different ZnO nanocrystals to NO_2_ gas under 365 nm light irradiation at room temperature. It can be seen that gas response increases when NO_2_ is injected, and then decreases rapidly to near the baseline as the NO_2_ is replaced by N_2_ within 10 min. The sensors are very sensitive to NO_2_ gas due to the large specific surface area of ZnO nanocrystals. Even at a very low gas concentration of 5 ppm, the sensors exhibit strong and stable signals compared to the baseline. Supplementary Fig. 10 presents plots of the gas-sensing response as a function of NO_2_ gas concentration. The response amplitude of these sensors increases with the NO_2_ gas concentration. The linear relationship suggests that the sensor response exhibits good dependence on the gas concentration. According to the definition of sensitivity (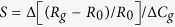), the sensitivities of the sensors below 25 ppm NO_2_ are about 0.75, 0.64 and 0.52 ppm^−1^ respectively, as displayed in Supplementary Fig. 10. ZnO-300/100–650/200 shows better gas sensing performance than our other ZnO nanocrystals, which is probably due to the reduced recombination of photogenerated electrons and holes from the high crystalline quality (or low C_bulk_/C_surface_) of ZnO-300/100–650/200.

## Discussion

According to the results obtained from the characterization analyses and evaluations of the photocatalytic properties above, a reasonable and comprehensive interpretation can be concluded on the mechanism for the enhanced photocatalytic performance of ZnO-300/100–650/200 (Fig. 5g). When ZnO catalyst is illuminated by light with energy matching or exceeding its band gap energy, the electrons in the valence band (VB) can be excited into the CB, leaving holes behind (path 1 in Fig. 5g). These excited electrons (including electrons from paths 1 and 2 in Fig. 5g) can react with dioxygen on the surface of ZnO catalyst to form superoxide radical anions (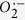) which subsequently undergo a series of reactions to produce reactive hydroxyl radicals (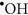) (path 7 in Fig. 5g). The holes in the VB ionize and oxidize the surface OH groups and water molecules to generate 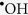 (path 7 in Fig. 5g). Finally, the dye molecules are oxidized by 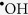 and holes into CO_2_, H_2_O and mineral acid (path 8 in Fig. 5g). For the photocatalytic systems mentioned in this paper, most of photogenerated charge carriers would recombine and eventually annihilate because of the large number of bulk defects in ZnO nanocrystals. Our work shows that reducing heating rate or employing a novel stepwise heat treatment process can improve the situation. This is attributed to two factors: a) both the reduction in bulk defects (or grain boundary defects) and the increase in surface oxygen vacancies are conducive to the separation and transfer of photo-generated carriers. In fact, the surface oxygen vacancies not only act as potential wells to trap either one or two electrons, but also facilitate the adsorbed O_2_ and H_2_O (or OH) in consuming electrons and holes (path 6 in Fig. 5g), whereas bulk defects increase their recombination rate (path 3 and 4 in Fig. 5g); (b) the larger specific surface area enhances light-harvesting, providing more photogenerated charge carriers for photocatalytic reactions. Compared with traditional heat treatment processes, the stepwise heat treatment process balances the specific surface area and the defect properties (concentration and spatial location) very well. Similarly, for NO_2_ gas sensing, suppressing the recombination of photogenerated carriers increases the number of surface-accumulated electrons; hence the charge carrier density is more sensitive to the surface adsorption conditions of nanomaterials. NO_2_ is a strong oxidizing gas that has become one of the most common air pollutants^58^. Upon exposure to NO_2_ gas, the photogenerated electrons that have migrated to the surface of ZnO nanocrystals will be rapidly captured by NO_2_ molecules (acting as an electron acceptor), absorbed in the surface active sites 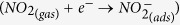. Thus, a depletion layer is created in the surface region of the ZnO nanocrystals due to the consumption of electrons, resulting in an increase in the resistance of a ZnO nanocrystal-based gas sensor.

Therefore, by virtue of the synergistic advantages of enhanced light-harvesting, effective separation of electron-hole pairs, ZnO-300/100–650/200 shows significant superior solar-driven photocatalytic activity and gas-sensing performance under UV light irradiation. The stability and recyclability of ZnO-300/100–650/200 is also investigated by employing the photodegradation of MO solution under simulated sunlight irradiation and the sensor response to NO_2_ under 365 nm light irradiation, respectively. As shown in Fig. 5c, the increased MO concentration is evacuated every 60 min in successive runs. After five cycles, no significant loss is observed. Figure 5e shows the reproducibility of the sensor based on ZnO-300/100–650/200 to 25 ppm NO_2_. As can be seen, the amplitude of the sensor responds to NO_2_ with no loss. As for the slight increase of response amplitude of the sensor with cycle number, this might be because adsorbed gas molecules do not completely desorb from the surface of ZnO nanocrystals. Furthermore, the photocatalytic activity of ZnO-300/100–650/200 under natural sunlight irradiation was also evaluated. As depicted in Supplementary Fig. 11, MO is completely decomposed within 120 min, and its degradation rate is about 0.18 times less than that under simulated sunlight irradiation. Several common gases – CH_4_, CO_2_, CO, H_2_S, NH_3_ and SO_2_ – were used as interfering gases at a concentration of 25 ppm to characterize the gas-sensing selectivity of the ZnO-300/100–650/200 based sensor (Fig. 5f). It is noted that we neglected the responses of the sensor to all gases other than NO_2._ These results demonstrate that ZnO nanocrystals obtained by the strategy in this work can serve well as an effective, recyclable photocatalyst or as a gas-sensitive material, with very high practicability.

In summary, a series of ZnO nanocrystal samples was prepared by a modified polymer-network gel method. Their crystallization was easily tuned by changing synthesis conditions. The stepwise heat treatment process used in this work can effectively improve the crystal quality of nanocrystals and suppress the aggregation of particles that usually results in reduction of the specific surface area. Under light irradiation, such well crystallized ZnO nanocrystals with large surface areas exhibit excellent photocatalytic and NO_2_ gas-sensing performance, both attributed to enhanced light-harvesting and effective separation of electron-hole pairs. Such synthesis strategies can be employed for preparation of other metal oxide nanocrystals with good crystallinity and high specific surface area, which are applicable in environmental remediation and monitoring.

## Methods

### Synthesis of ZnO nanocrystals

ZnO nanocrystals were synthesized using a modified polymer-network gel method. In a typical synthesis, 0.015 mol Zn(NO_3_)_2_·6H_2_O was slowly added into 50 ml deionized water under magnetic stirring to form a transparent solution. Then a given amount of tartaric acid was added into the solution in the molar 1.5:1 with respect to Zn(II) ion. Thereafter, 12 g glucose, 8.0773 g acrylamide (AM) and 1.6155 g *N*,*N*′-methylene-bisactylamide (MABM) monomers (AM/MABM mass ratio of 5:1) were successively added into Zn(II)-tartaric acid chelation solution while kept on stirring until fully dissolved. The resultant solution was heated to 90 °C on a hot plate to initiate the polymerization reaction, and a polyacrylamide gel was formed after a few minutes. A dark brown xerogel obtained by drying the gel at 120 °C for 24 h in a thermostat drier was ground into a fine powder in an agate mortar. Finally, the obtained powder was transferred into a muffle furnace, and annealed under different conditions in air to prepare various ZnO samples, denoted as ZnO-650/200, ZnO-650/400 and ZnO-300/100–650/200, respectively. The corresponding heat treatment procedures are given in Supplementary Table 3.

### Materials characterization

The X-ray diffraction (XRD) patterns were recorded on an X´pert pro MPD diffractometer with Cu Kα radiation (λ = 1.5418 Å and *θ* = 20–80°) at room temperature. The Brunauer-Emmett-Teller (BET) surface area was measured on a Micromeritics ASAP 2020 (USA) adsorption apparatus using N_2_ adsorption at −196 °C. Thermogravimetric (TG) and derivative thermogravimetric (DTG) analyses were carried out on a Netzsch-STA 449 C simultaneous thermal analyzer from 30 °C to 800 °C at a heating rate of 10 °C/min in air. The field emission scanning electron microscopy (FE-SEM) images were performed on a JEOL JSM-7500F (Japan) at an accelerating voltage of 15 kV. The morphologies of Transmission Electron Microscopy (TEM) and High-resolution TEM (HTEM) images were taken on a Tecnai G^2^ F20 microscope operating at an acceleration voltage of 200 kV. Photoluminescence (PL) spectra were obtained at room temperature by RF-5310pc fluorescence spectrophotometer with the excitation wavelength 325 nm. Ultraviolet-visible (UV-Vis) absorption spectra were obtained with an UNICAM-UV500 spectrophotometer to detect absorption of ZnO samples over the range of 300–800 nm. The X-ray photoelectron spectroscopy measurements were performed in a VG ESCALAB 210 (VG Scientific, UK) photoelectron spectrometer equipped with a Mg Kα_1,2_ exciting source and source power of 300 W. All the binding energies were calibrated with respect to the C *1 s* peak at 285.0 eV. Fourier Transform Infrared Spectra (FTIR) were recorded on an IR-200 spectrometer (Thermo Electron Co., US) in the frequency range of 400–4000 cm^−1^ with a resolution of 2 cm^−1^. The surface photovoltage (SPV) spectra were obtained by a instrument (Jilin University, China) consisting of a source of monochromatic light, a lock-in amplifier (SR830-DSP) with a light chopper (SR540), a photovoltaic cell and a computer at room temperature and in air atmosphere.

### Photocatalytic activity evaluation

The catalytic activity of the ZnO samples for the photodegradation of dyes were performed in a glass beaker with a volume of 250 ml under simulated sunlight irradiation by a 300 W Xe lamp. 50 mg of the ZnO photocatalysts were dispersed in 100 mL MO and RhB aqueous solution (4 mg/L), respectively. Before exposing the dispersions to light irradiation, they were ultrasonicated for 30 s, magnetically stirred for 5 min, and then kept in the darkness for 0.5 h to establish an adsorption-desorption equilibrium. Then the system was exposed to the simulated sunlight for photodegradation tests. At given time intervals, 4 mL aliquots were taken out and centrifuged (6000 rpm, 5 min) to remove the catalysts from the suspension. The residual concentration of MO or RhB in solution was analysed using a UV-Vis spectrophotometer. Moreover, the visible-light photocatalytic activity of the ZnO-300/100–650/200 samples for the degradation of MO and RhB were also performed by a 300 W Xe lamp with a 400 nm cutoff filter. The method was similar to the simulated sunlight degradation above mentioned.

For multi-cycle performance tests for the ZnO-300/100–650/200 samples, once the photocatalytic reaction of a testing cycle is complete, the subsequent cycle is then started after an aliquot of 4 mL of MO mother liquor (100 mg/L) and deionized water are added to the glass beaker to bring the concentration of the solution to approximately 4 mg/L. The length of each cycle was 100 minutes. In addition, the catalytic activity of the ZnO-300/100–650/200 samples for the photodegradation of MO was also performed under sunlight irradiation.

### Fabrication of sensor devices

500-nm-thick silicon dioxide (SiO_2_) passivated n-type Si (100) wafers were used as the substrates. A Ti (200-nm-thick)/Au (500-nm-thick) double-layer electrodes was deposited on the surface of SiO_2_ by thermal evaporation. Then the interdigital electrodes with a finger width of 50 μm and gap width of 50 μm were patterned by conventional photolithography and lift-off process. The sensors were fabricated by a simple spin-coating process. Firstly, the as-prepared ZnO nanocrystals were dissolved in ethanol to achieve a concentration of 3.0 mg/mL. Secondly, the ZnO suspension was spin-coated onto the interdigital electrodes at 500 rpm for 6 s and 3000 rpm for 30 s, respectively, followed by a drying at 80 °C for 8 h.

### Gas-sensitive performance evaluation

Gas-sensitive performance was measured with a home-made gas-sensing characterization system consisting of a test chamber equipped with UV-LED light sources (365 nm, 3 w), mass flow controller and Keithley 2700 source meter. The distance between the sensor and UV-LED was kept constant at 15 mm. During the measurements, the different concentration of NO_2_ gas diluted with N_2_ gas or pure N_2_ gas was periodical introduced into the test chamber at a total flow rate of 200 sccm. The gas feeding time of each cycle was fixed at 10 min for all tests. In addition, other gases were also tested to investigate the selectivity of the sensors. The real-time resistances of sensors were recorded by a PC with corresponding data acquisition hardware and software. All measurements were performed at room temperature and under continuous illumination. The sensing response in this paper was defined as 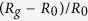, where *R*_*g*_ and R_0_ are the resistance of the ZnO films after exposing to target gas and nitrogen gas, respectively.

## Additional Information

**How to cite this article:** Zhang, Q. P. *et al*. A feasible strategy to balance the crystallinity and specific surface area of metal oxide nanocrystals. *Sci. Rep.* **7**, 46424; doi: 10.1038/srep46424 (2017).

**Publisher's note:** Springer Nature remains neutral with regard to jurisdictional claims in published maps and institutional affiliations.

## Supplementary Material

Supplementary Information

## Figures and Tables

**Table 1:** Average particle size, average crystal size and BET for different MOS nanocrystals.

Nanocrystals		Crystal phase	Average particle size (nm)	Average crystal size (nm)	BET surface area (m^2^/g)
ZnO	ZnO-650/200	wurtzite phase	100 ± 5	42.13	29.9706
	ZnO-650/400		90 ± 5	42.40	17.6076
	ZnO-300/100–650/200		90 ± 5	41.40	29.3528
	CeO_2_	fluorite cubic phase	35 ± 5	16.38	60.0477
	ZrO_2_	monoclinic/tetragonal mixed phase	50 ± 5	17.17	39.1464
	α-Fe_2_O_3_	rhombohedral phase	100 ± 5	44.33	47.3008
	SnO_2_	tetragonal rutile phase	200 ± 5	42.07	32.9202
	TiO_2_	anatase/rutile mixed phase	30 ± 5	28.55	71.0399
