# Supplementary material for: A feasible strategy to balance the crystallinity and specific surface area of metal oxide nanocrystals
Source: Sci Rep. 2017 Apr 24;7:46424. doi: 10.1038/srep46424 (PMC5402303; doi:10.1038/srep46424)
Supplement: Supplementary Information [file srep46424-s1.pdf]

Supplementary Information

A feasible strategy to balance the crystallinity and specific surface of metal oxide nanocrystals

Q. P. Zhang[1], X. N. Xu[1], Y. T. Liu[1], M. Xu[1,*], S. H. Deng[1], Y. Chen[1], H. Yuan[1], F. Yu[1], Y. Huang[1], K. Zhao[1], S. Xu[2], G. Xiong[3,*]

[1] Key Laboratory of Information Materials of Sichuan Province & School of Electrical and Information Engineering, Southwest University for Nationalities, Chengdu 610041, China

[2] Plasma Sources and Application Center, NIE, and Institute of Advanced Studies, Nanyang Technological University, 637616, Singapore

[3] Department of Chemistry, University of Cambridge, CB2 1EW, Cambridge, UK

[*] Electronic-mail: hsuming_2001@aliyun.com (M. Xu), gx206@cam.ac.uk (G. Xiong)

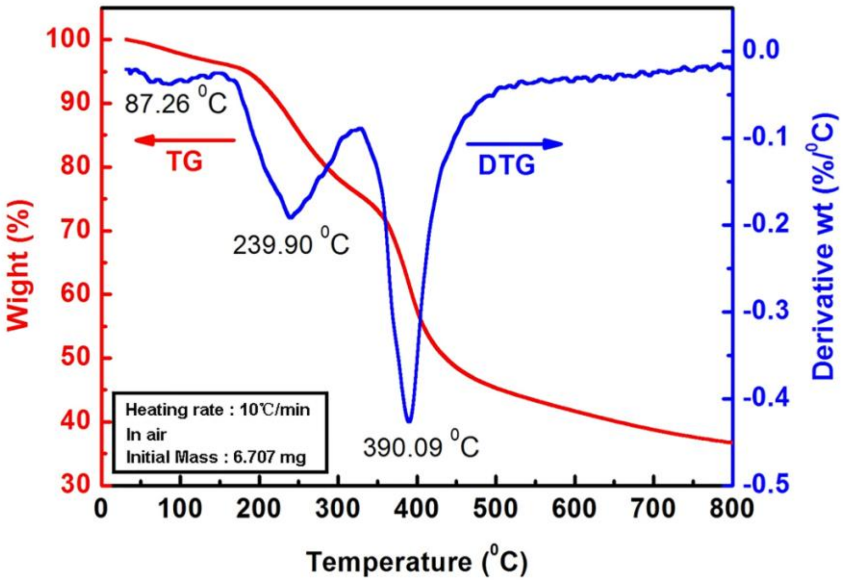

Supplementary Figure 1. TG/DTG curves of ZnO xerogel powder prepared using a modified polymer-network gel method.

In order to determine the stepwise heat treatment strategy，a thermogravimetric analysis is carried out. Supplementary Figure 1 is the annealing behavior of ZnO xerogel powder in air. As can be seen from the figure, there were four weight loss stages: (1) Before 140℃, a weight loss of 3.26% was caused by the evaporation of absorbed water. (2) and (3) From 140℃ to 480℃, the large weight loss of 50.39% could be attributed to decomposition and combustion of organic compounds (such as, tartaric acid, glucose and polyacrylamide). The decomposition temperature of the two stage is 239.90 and 390.09℃, respectively. At such stage, the quality of ZnO nanocrystals is influenced by the chemical bond rupture and thermal stress. (4) After 480℃, a weight loss of 9.65% was due to the decomposition of residues and the loss of surface atoms. It was obvious that the organic precursor was almost burned out at 550℃, resulting in the formation of ZnO powder. Higher temperature may cause more surface oxygen atoms to be lost[1]. Using the standard Kröger-Vink notation, the formation of oxygen vacancies at elevated temperatures can be described by the following equilibrium[2]:

$$O_o \leftrightarrow V_O^{\cdot\cdot} + 2e' + \frac{1}{2}O_2$$

Where O_O and $V_O^{\cdot\cdot}$ denote the lattice oxygen and the oxygen vacancies, respectively. As mentioned above, the features of ZnO nanocrystals can be easily tuned by selecting a proper heat treatment route, and thus optimizing heat treatment process is a simple and easy method to improve the certain properties of materals.

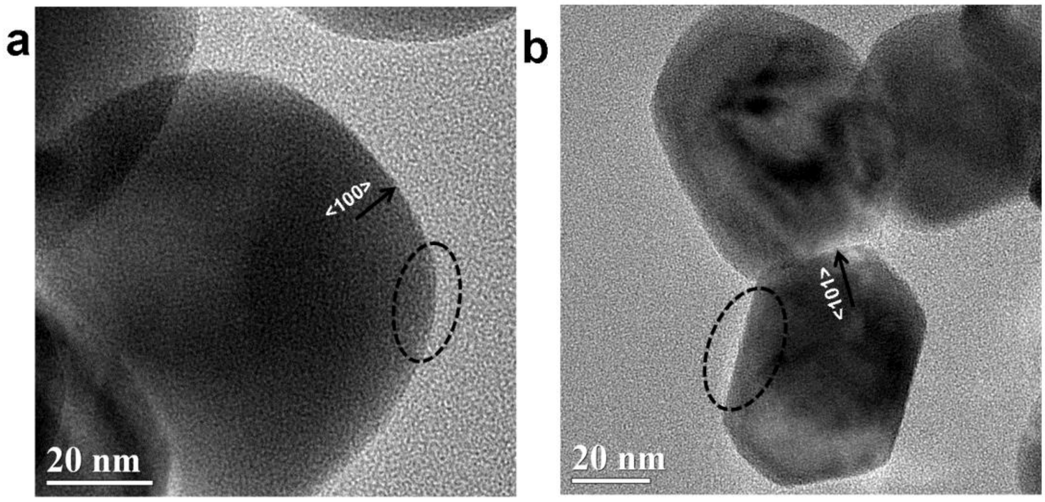

Supplementary Figure 2. TEM images of different ZnO nanocrystals: (a) ZnO-650/200; (b) ZnO-300/100-650/200. HRTEM images shown in Fig.1 derive from the region surrounded by dotted lines.

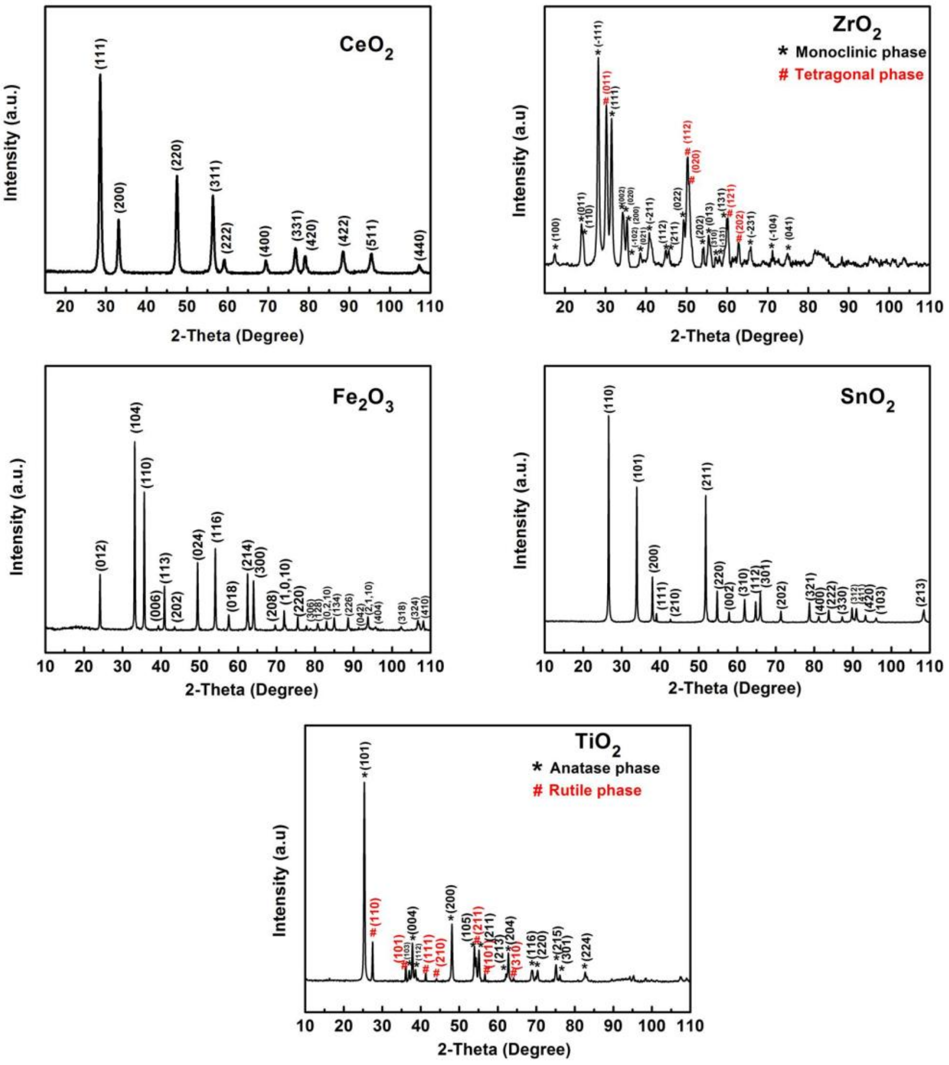

Supplementary Figure 3. XRD patterns of other metal oxide nanocrystals prepared in ways similar to the synthesis of ZnO-300/100-650/200.

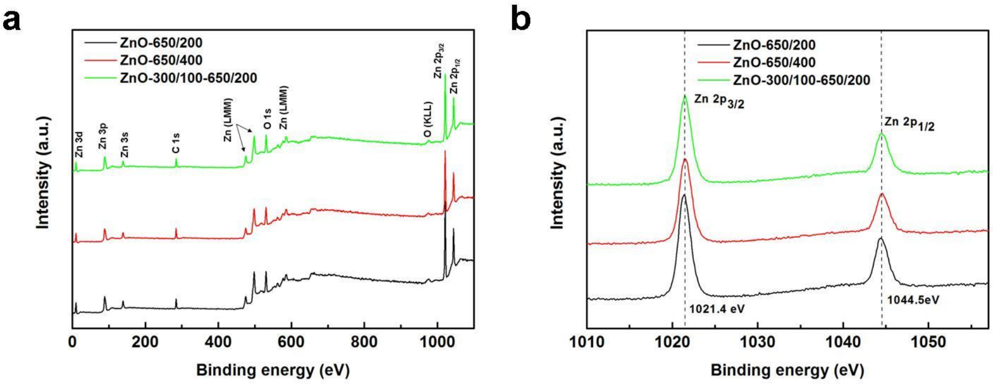

Supplementary Figure 4. (a) XPS survey spectra and (b) Zn 2p XPS spectra of ZnO nanocrystals obtained using different heat treatments.

Supplementary Figure 4a is the XPS survey spectrum of the different ZnO nanocrystals. It is evident that these nanocrystals have consistent photoelectron lines at same binding energy values, they are ascribed to Zn 3d, Zn 3p, Zn 3s, C1s, O1s, and Zn 2p core levels and to Zn *LMM* and O *KLL* Auger features. It shows the ZnO nanocrystals consisting of element Zn and O, without evident impurities. The presence of C is likely attributed to the unpredictable contamination resulting from sample handling. The corresponding high resolution Zn 2p spectra shows its emission is split obviously into two symmetric peaks (Supplementary Fig. 4b). The peaks located at binding energies of 1021.2±0.3 eV was attributed to the Zn $2p_{3/2}$ and the other one at binding energies of 1044.5±0.5 eV to Zn $2p_{1/2}$, indicating the presence of Zn^{2+} lattice ions in all ZnO catalysts[3,4]. In addition, ZnO-650/200 exhibit more intense Zn 2p XPS peak and a tiny chemical shift of the peak toward low binding energy compared to ZnO-650/400 and ZnO-300/100-650/200, suggesting that more Zn atoms were bound to O atom[5].

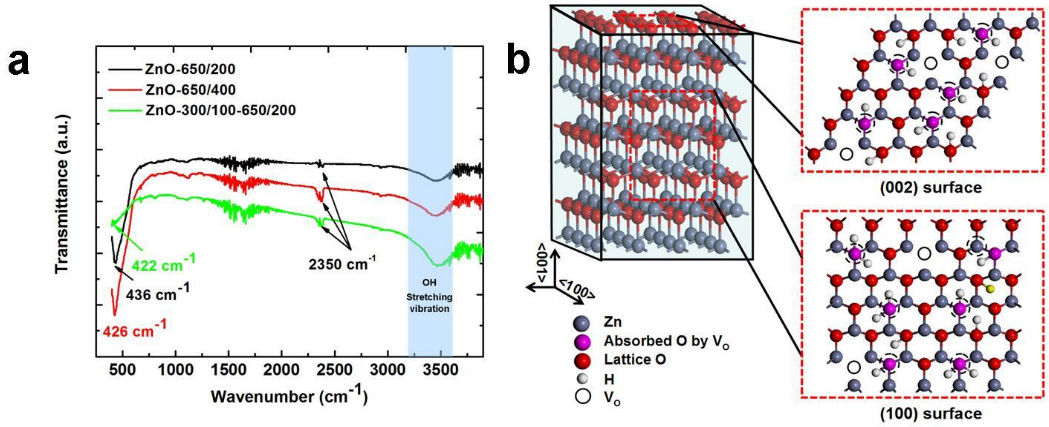

Supplementary Figure 5. (a) FTIR spectra of ZnO nanocrystals obtained using different heat treatments. (b) Schematic diagram of ZnO (002) and (100) surfaces with OH groups and oxygen vacancies. On the O-ZnO (002) surface, the OH groups form only through dissociation of water on oxygen vacancy sites, whereas the partial dissociation of water on the ZnO (100) surface yields coexistent H_2O and OH groups[6].

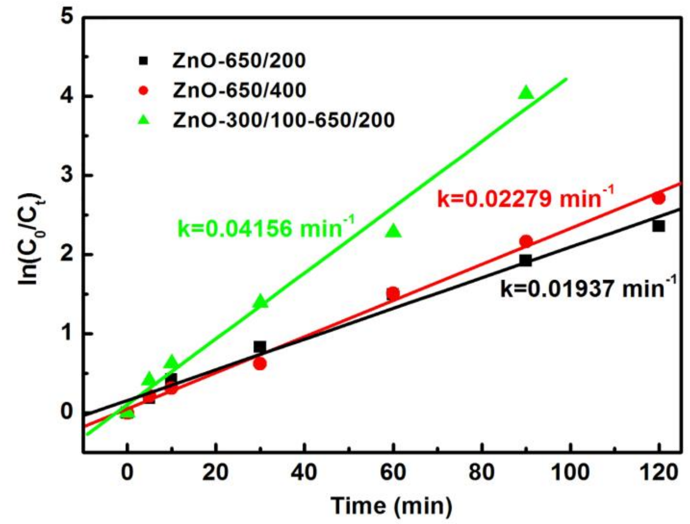

Supplementary Figure 6. First-order linear transforms quantifying the decomposition of MO over ZnO nanocrystals under simulated sunlight irradiation.

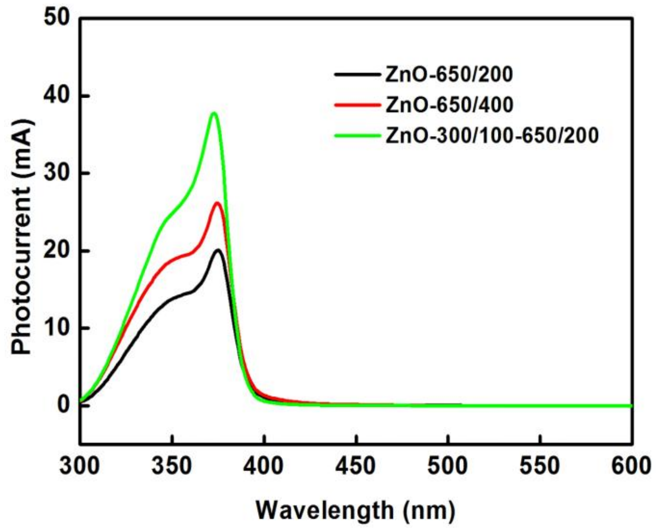

Supplementary Figure 7. SPC spectra of ZnO nanocrystals obtained using different heat treatments.

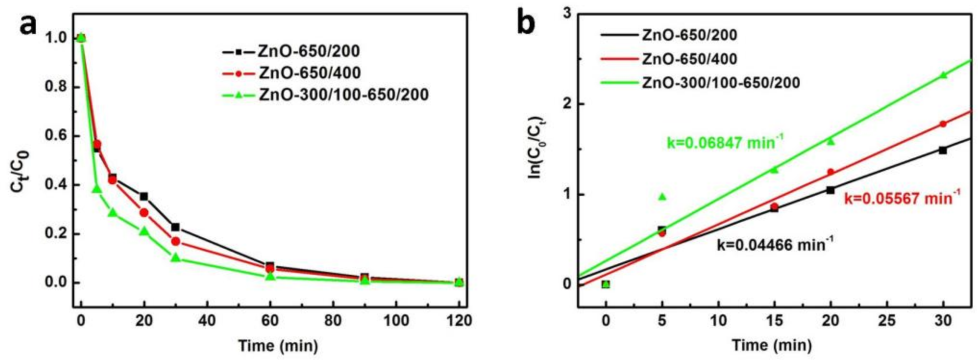

Supplementary Figure 8. (a) Photodegradation of RhB over ZnO nanocrystals under simulated sunlight irradiation and (b) the corresponding pseudo first-order kinetics curve. The normalized concentration of RhB aqueous solution is monitored by measuring absorbance at 552 nm.

The simulated sunlight-induced photocatalytic activity of different ZnO nanocrystals on photodegradation of RhB are compared, as shown in Supplementary Fig. 8. It can be seen that the photodegradation of RhB is fitted to pseudo-first-order kinetic model. The photocatalytic activity trend follows the order: ZnO-300/100-650/200 > ZnO-650/400 > ZnO-650/200.

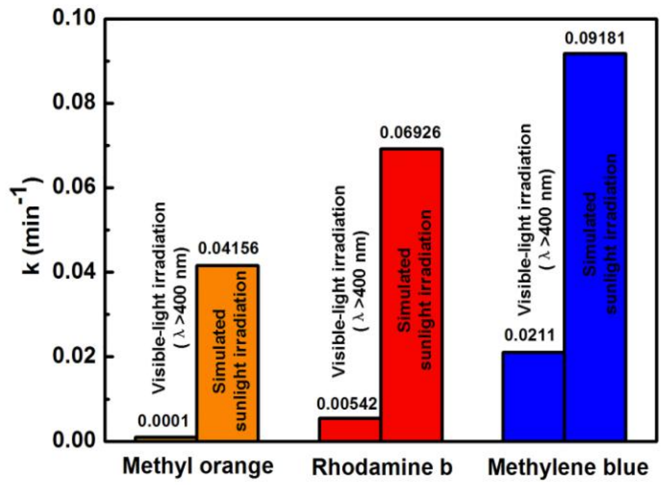

Supplementary Figure 9. Apparent rate constants for the photocatalytic decomposition of MO, RhB and MB over ZnO-300/100-650/200 under different light irradiation.

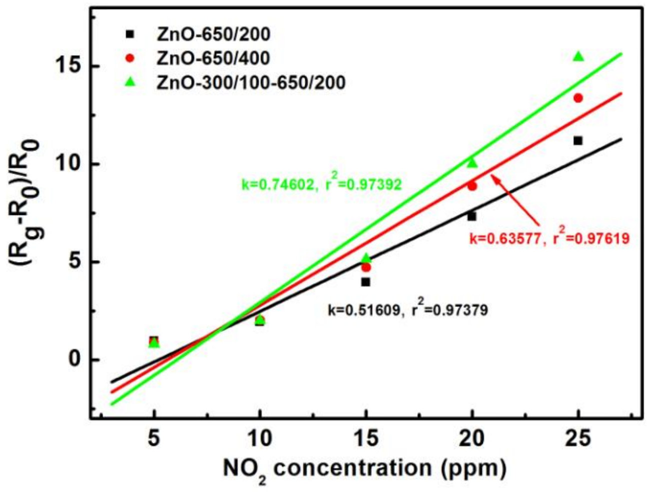

Supplementary Figure 10. Sensitivity of sensors based on different ZnO nanocrystals to 5-25 ppm of NO_2 under 365 nm light irradiation at room temperature.

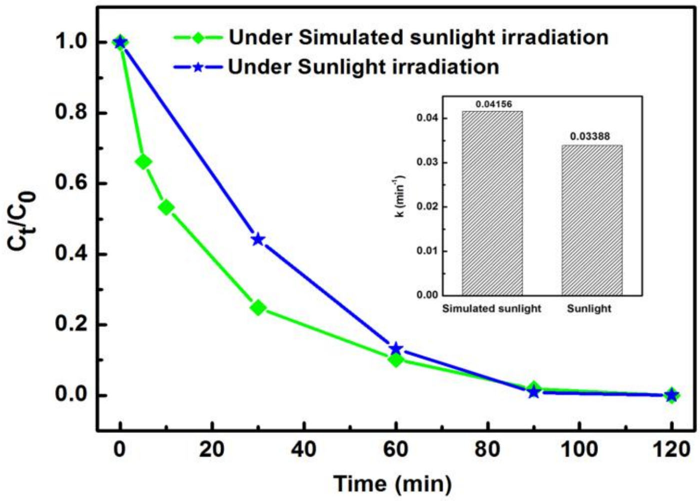

Supplementary Figure 11. Comparison of photodegradation of MO over ZnO-300/100-650/200 under simulated and natural sunlight irradiation. Inset shows the corresponding apparent rate constants.

Supplementary Table 1. Comparison between the nanocrystals prepared by this strategy and those by other reported methods.

Nanocrystals	Method	Annealing mode	Average particle size (nm)	BET surface area (m^2/g)	Ref.
ZnO	Flame-spray pyrolysis	One-step (750℃)	57.7	12.0	[7]
	Solvothermal	One-step (400℃)	64.9	16.5	[8]
	Sol-gel	One-step (600℃)	70±5	14.2	[9]
	Modified polymer-network gel	Stepwise (300℃ -650℃)	90±5	29.4	This work
TiO_2	Tow-step sol-gel	One-step (450℃)	18.6	74.5	[10]
	Sol-gel	One-step (450℃)	15.0	73.7	[11]
	Modified polymer-network gel	Stepwise	30±5	71.0	This work
SnO_2	Hydrothermal	-	200	18.7	[12]
	Surfactant-assisted solvothermal	One-step (750℃)	12.0	35.0	[13]
	Modified polymer-network gel	Stepwise	200±5	32.9	This work
CeO_2	Hydrothermal	One-step (550℃)	30±5	32.8	[14]
	Vapor-to-solid	One-step (350℃)	30.0	26.2	[15]
	Modified polymer-network gel	Stepwise	35±5	60.0	This work
ZrO_2	Chemical co-precipitation	One-step (550℃)	18.0	42.6	[16]
	Solution combustion	One-step (500℃)	31.2	34.5	[17]
	Modified polymer-network gel	Stepwise	50±5	39.1	This work
α-Fe_2O_3	PVP-assisted solvothermal	-	50.0	18.3	[18]
	Non-ionic soft template	One-step (500℃)	30±5	44.4	[19]
	Modified polymer-network gel	Stepwise	100±5	47.3	This work

Supplementary Table 2. Comparison of XPS data for oxygen and zinc at the surface of different ZnO nanocrystals

Samples	Bing energy of oxygen (eV)		The atomic ratio of O_L to O_H	The atomic ratio of Zn to O_L
	O_L	O_H		
ZnO-650/200	530.20	531.75	1.54	1.504
ZnO-650/400	530.35	531.85	1.42	1.570
ZnO-300/100-650/200	530.25	531.75	1.32	1.775

Supplementary Table 3. The heat treatment procedures of samples ZnO-650/200, ZnO-650/400 and ZnO-300/100-650/200.

Samples	Pre-calcination (300℃)		Recalcination (650℃)		Cooling mode
	Temperature rising time	Calcining time	Temperature rising time	Calcining time	
ZnO-650/200	-	-	200	300	N*
ZnO-650/400	-	-	400	300	N*
ZnO-300/100-650/200	100	100	200	200	N*

* Represents the natural air cooling.

Supplementary References

1. Jing, L., Xu, Z., Sun, X., Shang, J. & Cai, W. The surface properties and photocatalytic activities of ZnO ultrafine particles. *Appl. Surf. Sci.* **180**, 308-314 (2001).
2. Pan, X., Yang, M. Q., Fu, X., Zhang, N. & Xu, Y. J. Defective TiO_2 with oxygen vacancies: synthesis, properties and photocatalytic applications. *Nanoscale* **5**, 3601-3614 (2013).
3. Bai, L. N., Wang, S., Sun, H. M., Jiang, Q. & Lian, J. S. Disordered ZnO nanoparticles with extremely intense deep-level emission and enhanced photocatalytic activity. *Appl. Surf. Sci.* **313**, 888-895 (2014).
4. Uddin, M. T. et al. Nanostructured SnO_2–ZnO heterojunction photocatalysts showing enhanced photocatalytic activity for the degradation of organic dyes. *Inorg. Chem.* **51**, 7764-7773 (2012).
5. Zhang, Y. et al. X-ray photoelectron spectroscopy study of ZnO films grown by metal-organic chemical vapor deposition. *J. Cryst. Growth* **252**, 180-183 (2003).
6. Noei, H. et al. The identification of hydroxyl groups on ZnO nanoparticles by infrared spectroscopy. *Phys. Chem. Chem. Phys.* **10**, 7092-7097 (2008).
7. Mekasuwandumrong, O., Pawinrat, P., Praserthdam, P. & Panpranot, J. Effects of synthesis conditions and annealing post-treatment on the photocatalytic activities of ZnO nanoparticles in

the degradation of methylene blue dye. *Chem. Eng. J.* **164**, 77-84 (2010).
8. Becker, J., Raghupathi, K. R., St. Pierre, J., Zhao, D. & Koodali, R. T. Tuning of the crystallite and particle sizes of ZnO nanocrystalline materials in solvothermal synthesis and their photocatalytic activity for dye degradation. *J. Phys. Chem. C* **115**, 13844-13850 (2011).
9. Shidpour, R., Simchi, A., Ghanbari, F. & Vossoughi, M. Photo-degradation of organic dye by zinc oxide nanosystems with special defect structure: Effect of the morphology and annealing temperature. *Appl. Catal. A-Gen.* **472**, 198-204 (2014).
10. Lee, S. et al. Two-step sol-gel method-based TiO_2 nanoparticles with uniform morphology and size for efficient photo-energy conversion devices. *Chem. Mater.* **22**, 1958-1965 (2010).
11. Yu, Y. et al. The design of TiO_2 nanostructures (nanoparticle, nanotube, and nanosheet) and their photocatalytic activity. *J. Phys. Chem. C* **118**, 12727-12733 (2014).
12. Han, X. et al. Synthesis of tin dioxide octahedral nanoparticles with exposed high-energy {221} facets and enhanced gas-sensing properties. *Angew. Chem.* **121**, 9344-9347 (2009).
13. Chu, D. et al. Enhanced properties of SnO_2 nanocrystals with decreased size for ppb-level acetaldehyde decomposition. *ChemCatChem* **3**, 371-377 (2011).
14. Lei, W. et al. Surface-structure sensitivity of CeO_2 nanocrystals in photocatalysis and enhancing the reactivity with nanogold. *ACS Catal.* **5**, 4385-4393 (2015).
15. Rajendran, S. et al. Ce^{3+}-ion-induced visible-light photocatalytic degradation and electrochemical activity of ZnO/CeO_2 nanocomposite. *Sci. Rep-UK* **6**, 31641 (2016).
16. Bansal, P., Chaudhary, G. R. & Mehta, S. K. Comparative study of catalytic activity of ZrO_2 nanoparticles for sonocatalytic and photocatalytic degradation of cationic and anionic dyes. *Chem. Eng. J.* **280**, 475-485 (2015).
17. Singhania, A. & Gupta, S. M. Nanocrystalline ZrO_2 and Pt-doped ZrO_2 catalysts for low-temperature CO oxidation. *Beilstein J. Nanotech.* 8, 264-271 (2017).
18. Zheng, Y. et al. Quasicubic α-Fe_2O_3 nanoparticles with excellent catalytic performance. *J. Phys. Chem. B* **110**, 3093-3097 (2015).
19. Park, C., Jung, J., Lee, C. W. & Cho, J. Synthesis of mesoporous α-Fe_2O_3 nanoparticles by non-ionic soft template and their applications to heavy oil upgrading. *Sci. Rep-UK* **6**, 39136 (2016).
